# Human leukocyte antigen-haploidentical donor-derived cytokine-induced killer cells are safe and prolong the survival of patients with advanced non-small cell lung cancer

**DOI:** 10.3892/ol.2014.2558

**Published:** 2014-09-24

**Authors:** SHIYONG WANG, HUI ZHANG, CHANG LIU, XUE JIAO, DIJIE LIU, WEILI DU, YING HE, ZHE ZHANG, XIUYAN WU, JIALING WANG, CHUNYAN LIANG, LU ZHANG, SHU LIU

**Affiliations:** Department of Biotherapy and Laboratory of Biotherapy, The Fourth Affiliated Hospital of China Medical University, Shenyang, Liaoning 110032, P.R. China

**Keywords:** cytokine-induced killer cells, immunotherapy, adoptive, allogeneic, malignant tumor, carcinoma, non-small cell lung

## Abstract

The aim of the present study was to evaluate the safety and efficacy of administering cytokine-induced killer cells (termed allogeneic CIKs), obtained from the blood of the offspring of patients, for the treatment of non-small cell lung cancer. Symptoms, signs and laboratory assessment results for 303 cancer patients were collected prior to and following treatment with autologous or allogeneic CIKs. In addition, 54 patients with advanced non-small cell lung cancer (NSCLC) were enrolled and divided into allogeneic CIK and optimal support groups (n=27 per group) according to gender, age, Karnofsky performance status score, TNM stage and histological type. In addition, overall survival (OS) was compared between the two groups. A total of 303 patients were treated with CIKs for 647 cycles, with 308 and 339 cycles in the autologous and allogeneic CIK groups, respectively. The mean number of CIKs in the autologous and allogeneic groups was 2.11±0.32×10^10^ and 2.29±0.36×10^10^, respectively, with no marked differences identified between the two groups (t=1.147; P>0.05). The predominant adverse events included insomnia, fever, nausea, vomiting and mild abdominal pain, which were found, respectively, in nine (6.8%), eight (6.0%), two (1.5%) and one (0.8%) patients receiving autologous CIKs and 11 (6.5%), 10 (5.9%), one (0.6%) and one (0.6%) patients receiving allogeneic CIKs, with no marked differences identified between the two groups (P>0.05). Adverse events were not associated with cell count, frequency or duration of treatment. Following CIK treatment, the outcomes of routine blood tests, and liver and kidney function tests, as well as immune function and electrocardiogram examinations remained unchanged (P>0.05). The median OS was 11.0 months (95% confidence interval (CI), 8.6–13.4 months) and 8.0 months (95% CI, 5.3–10.7 months) for NSCLC patients receiving allogeneic CIKs and optimal support, respectively; a statistically significant difference was identified (χ^2^=5.618; P=0.018). The present study demonstrated that CIKs from human leukocyte antigen haploidentical donors are safe and prolong the survival of NSCLC patients.

## Introduction

Autologous cytokine-induced killer cells (CIKs) are a safe and effective form of immunotherapy for malignant tumors (1–4). Previous studies have found that CIK therapy in combination with chemotherapy, radiotherapy and surgery significantly increases progression-free, disease-free and overall survival (OS), which are closely associated with the frequency or cycle count of CIK immunotherapy (5–7). However, few studies have investigated the translation of prolonged progression-free survival, time to progression and disease-free survival into survival benefit (8–11). Thus, further investigation is required regarding the proper selection of donors and recipients, the effective *in vitro* induction methods, the timing and route of infusion, the appropriate number of CIKs for infusion, and the combination of CIKs with other treatments (12).

In China, treatment with dendritic cells, CIKs and natural killer cells has been included in the medical insurance system, which ensures that patients can afford such treatments. As practitioners and investigators in CIK therapy, the present study failed to convince other physicians, patients and their relatives that CIK treatment is an effective strategy for the early treatment of cancer. Currently, CIK therapy remains the last resort for cancer patients, when patients have exhausted the existing therapeutic methods. Consequently, CIK therapy is often considered for terminally ill cancer patients, which places CIK therapy in a predicament and vicious cycle. For advanced cancer patients (who have undergone numerous treatment modalities) or those who are terminally ill, autologous CIK therapy is limited in its efficacy or is infeasible, as CIKs barely proliferate, and the number and function of peripheral blood-induced CIKs do not meet the requirements of immunotherapy. Secondly, the separation of several thousand milliliters of peripheral blood using blood cell separators is poorly tolerated by patients. Furthermore, the ‘vacuum period’, between the separation of mononuclear cells from the peripheral blood and the infusion of CIKs back into the body, may take approximately two weeks. Thus, for cancer patients who have undergone multiple treatment modalities, it is necessary to identify alternative donors for CIKs and an optimal selection is the offspring of cancer patients.

Healthy donors are often unwilling to provide several thousand milliliters of peripheral blood for the isolation of mononuclear cells. However, several previous studies, including the present study, have developed a method for the collection of CIKs (13). In brief, 50–100 ml of peripheral blood is collected and cultured for approximately three weeks to yield ~1×10^10^ of CIKs. With this method, the proportion of CD3^+^CD56^+^ cells is *>2*5%, which meets the treatment requirements. In the present study, CIKs were collected from the peripheral blood obtained from the adult offspring of cancer patients, and the safety and efficacy of administering CIKs to patients for the treatment of cancer were evaluated.

## Patients and methods

### Clinical data

A total of 303 inpatients with malignant tumors who received CIK treatment were recruited from the Department of Biotherapy, the Fourth Affiliated Hospital of China Medical University (Shenyang, China) between January 2008 and December 2011. Of these patients, 133 received treatment with CIKs obtained from an autologous blood source (the autologous group) and 170 underwent treatment with allogeneic CIKs obtained from the blood of human leukocyte antigen (HLA) haploidentical donors (the allogeneic group). The demographic and disease characteristics of the patients are shown in Table I. Symptoms, signs and the outcomes of routine blood tests, and kidney and liver function tests, as well as electrocardiographic examinations were compared prior to and following the CIK infusion. In addition, adverse events were recorded using the National Cancer Institute Common Terminology Criteria for Adverse Events version 3.0 (2003). An evaluation of immune function identified that CD3^+^, CD3^+^CD4^+^, CD3^+^CD8^+^, CD3^−^CD56^+^CD16^+^ and CD19^+^ cells, and IgG, IgM, IgA, C3 and C4 were routinely detected in the peripheral blood. Prior to treatment, written informed consent was obtained from patients and their relatives, and the possible adverse events were explained. The entire protocol was approved by the Ethics committee of the Fourth Affiliated Hospital of China Medical University.

### CIK induction and intravenous infusion

Venous blood was collected prior to chemotherapy or at least one month following chemotherapy. For the offspring (HLA haploidentical donors), the routine blood tests, and liver and kidney function tests were normal and negative for hepatitis A virus-IgM, hepatitis B surface (HBs) antigen (Ag), HBs antibody (Ab), hepatitis B e (HBe)-Ag, HBe-Ab, hepatitis B core-Ab, hepatitis C virus (HCV)-Ab, HCV-IgG, Syphilis-Ab and human immunodeficiency virus-Ab. As reported in our previous study (13), 50 ml of peripheral venous blood was collected and the mononuclear cells were isolated using Lymphocyte Separation Medium (Haoyang Biological Manufacture, Co., Ltd., Tianjin, China). These cells were added to flasks precoated with RetroNectin (Takara, Shiga, Japan) and CD3 monoclonal antibodies (Johnson & Johnson, New Brunswick, NJ, USA) followed by incubation in serum-free KBM551 (Takara) at 37°C in a 5% CO_2_ atmosphere. On the day of culture, 1,000 U/ml human recombinant interferon-γ (PeproTech, Rocky Hill, NJ, USA), 500 U/ml recombinant human interleukin (rhIL)-1α (Invitrogen Life Technologies, Carlsbad, CA, USA) and 1,000 U/ml rhIL-2 (Quangang Pharmaceutical Co., Ltd., Jinan, China) were added. Four days later, 1,000 U/ml rhIL-2 was added and the cells were transferred into a GT-T610 culture bag (Takara). Cell growth was observed every other day and cells were stained with 0.4% trypan blue (Yocon Biotechnology Company, Co., Ltd., Beijing, China) and the viable cells were counted. Following 18 days of culture, cells were infused once daily (>1×10^9^ cells; viability rate, >95%). A cycle of treatment comprised of between three and five infusions and >5×10^9^ cells were infused per cycle. Prior to infusion, the surface immunophenotype of CIKs was determined by a standard fluorescence cytometry labeling protocol using fluorochrome-conjugated monoclonal mouse anti-human antibodies against CD3, CD8, CD4, CD45, CD19 and CD16CD56 (BD Biosciences, San Jose, CA, USA). Assays for the detection of bacteria, mildew and endotoxin were performed. Briefly, the presence of bacteria and mildew was investigated by incubating cultured CIKs on agar at 37°C, with subsequent inspection for the growth of microbial contaminants. Endotoxin levels were determined using a chromogenic endotoxin assay kit (Chinese Horseshoe Crab Reagent Manufactory, Co., Ltd., Xiamen, China), according to the manufacturer’s instructions. Only sterile and endotoxin-free preparations were used clinically.

### Evaluation of survival following treatment with allogeneic CIKs

The paired method was performed on the patients with pathologically confirmed stage IIIb-IV non-small cell lung cancer (NSCLC). Clinical data, including name, gender, age, Karnofsky performance status (KPS), pathological type (14), TNM stage (15) and history of treatment, were collected. These patients had a KPS score of ≥60 and a predicted survival of more than three months. Patients were divided into the following two groups: The CIK group and the optimal support group. In the CIK group, patients were treated with allogeneic CIKs for at least two cycles. Observations were performed from the initial therapy until mortality or until the final follow-up, and survival was determined. In the optimal support group, patients receiving optimal support therapy in the interim between receiving CIK infusions were recruited, and gender, age, KPS score and disease stage were matched between the two groups. The follow-up was performed via telephone or hospital visit.

### Statistical analysis

SPSS software package version 13.0 (SPSS, Inc., Chicago, IL, USA) was employed for statistical analysis. Quantitative data were expressed as the mean ± standard deviation and comparisons were performed using Student’s t-test. Qualitative data were compared using the χ^2^ and Fisher’s exact tests. Survival was delineated by the Kaplan-Meier method, followed by the log-rank test and P<0.05 was considered to indicate a statistically significant difference.

## Results

### Safety evaluation of CIK therapy

The general information of the patients with malignancies who received CIK therapy is shown in Table I. Age, gender and histological type were comparable between the two groups and a total of 647 cycles were applied (n=308 in the autologous group and n=339 in the allogeneic group). The total number of CIKs used for therapy in the autologous group was between 1.76×10^10^ and 1.35×10^11^ (mean, 2.11±0.32×10^10^) and between 1.16×10^10^ and 6.71×10^10^ (mean, 2.29±0.36×10^10^) in the allogeneic group, with no marked differences identified between the two groups (t=1.147; P>0.05). The majority of patients received less than four cycles of therapy (Table II) and the therapy was completed within one year. In 13 and four patients, therapy endured for more than one and two years, respectively. In addition, four patients received >10 cycles of therapy (autologous group, n=3; allogeneic group, n=1). In the autologous group, the highest number of therapy cycles was 20 for one patient, with therapy lasting four years with a total of 1.35×10^11^ CIKs. In addition, one patient underwent 13 cycles of therapy, which was completed in two years with a total of 7.3×10^10^ CIKs and one patient received 11 cycles of therapy, which was completed over 2.5 years with a total of 6.5×10^10^ CIKs. In the allogeneic group, one patient received 11 cycles of therapy, which was completed over 3.5 years with a total of 6.71×10^10^ CIKs.

During and following the infusion of CIKs, among the 133 patients in the autologous group and 170 patients in the allogeneic group, euphoria was observed in 66 (49.6%) and 86 (50.6%) patients, physical improvements in 21 (15.8%) and 28 (16.5%) patients, and pain relief in 15 (11.3%) and 20 (11.8%) patients, respectively. The major adverse events included insomnia, fever, nausea, vomiting and mild abdominal pain, which were identified in nine (6.8%), eight (6.0%), two (1.5%) and one (0.8%) patients, respectively, in the autologous group and 11 (6.5%), 10 (5.9%), one (0.6%) and one (0.6%) patients, respectively, in the allogeneic group. No marked differences in adverse events were identified between the two groups (P>0.05). Adverse events usually occurred on the day of infusion. Insomnia was non-responsive to diazepam and spontaneously recovered between three and five days later. Fever was mild or moderate, body temperature was ≤38.5°C and concomitant flu-like symptoms were present; the fever recovered spontaneously between one and three days later. Nausea, vomiting and mild abdominal pain were rarely observed in these patients and when present, the patients often recovered following symptomatic treatment. Symptoms of allergic reaction or rejection, such as rashes, diarrhea, jaundice and hematuria, were rare, with rashes observed in only one patient. These adverse events were not associated with the number of CIKs used for therapy, the frequency of the therapy, chronic rejection or accelerated rejection due to repeated infusions. Notably, after two months, no evident rejection was observed in the four patients who received CIKs from the blood of two of their offspring. In addition, the outcomes of routine blood tests and liver and kidney function tests, as well as electrocardiogram examinations were compared between the two groups, prior to and following therapy within each group, and no significant differences were identified (P>0.05; Table III). Data for the autologous group are not shown.

To determine whether the patients’ stage was associated with the occurrence of adverse events, 94 patients with lung cancer (Table I) that were treated with CIKs were divided according to their TNM stage, and the adverse events were compared. Among the 40 patients in the autologous group, 10 patients with stage I–IIIa disease developed fever, insomnia and flu-like symptoms, and 30 patients with stage IIIb-IV disease developed nausea, vomiting, fever and flu-like symptoms. Of the 54 patients in the allogeneic group, seven patients with stage I–IIIa disease developed fever, insomnia, rashes and mild abdominal pain, and 47 patients with stage IIIb-IV disease developed nausea, vomiting, fever and flu-like symptoms. No marked differences were observed in adverse events between stage I–IIIa and IIIb-IV patients within the same therapy group, and between patients in the same TNM stage but different therapy group (P>0.05).

**Figure 1:**
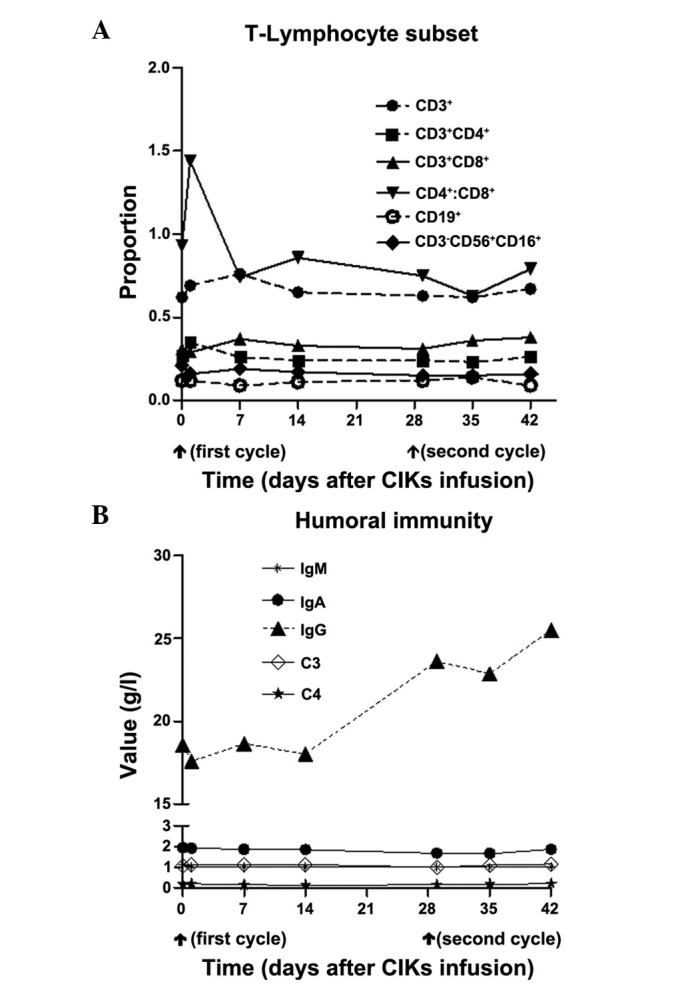
Dynamic change of immune function in four cancer patients following a high-dose CIK treatment. Four cancer patients (stable disease) were treated with an intravenous infusion of CIKs for two cycles only (>1×10^10^ per cycle for 28 days). (A) Lymphocyte subset percentage and (B) humoral immune function were detected prior to and two, seven and 14 days following the CIK infusion, for each cycle. CIK, cytokine-induced killer cell.

With regard to immune function, cellular and humoral immune function were comparable between the patients in the two groups prior to and following treatment (P>0.05; Table IV). To evaluate the impact of high-dose short-term infusion of CIKs on immune function, four patients (one in the autologous group and three in the allogeneic group) with KPS scores of >90 and stable disease were recruited and treated only with intravenous infusion of CIKs for two cycles (>1×10^10^ per cycle for 28 days). Immune function was detected prior to and two, seven and 14 days following the infusion (Fig. 1). On day two of the first cycle of CIK infusion, the proportion of CD3^+^, CD3^+^CD4^+^ and CD3^+^CD8^+^ cells, and the CD4^+^/CD8^+^ ratio were increased, which was most evident for the increasing ratio of CD4^+^/CD8^+^ (Fig. 1A). However, on day seven, the above indexes returned to levels, which were comparable with or slightly lower than the levels prior to infusion. This condition continued into day 14 following the second cycle of CIK infusion. IgG levels continued to increase over two consecutive cycles of CIK infusion (Fig. 1B). The proportion of CD19^+^ and CD3^−^CD56^+^CD16^+^ (Fig. 1A), and humoral immunity indexes, IgA, IgM, C3 and C4 (Fig. 1B), were almost invariant over two consecutive cycles of CIK treatment.

### Survival of patients with advanced NSCLC following treatment with allogeneic CIKs

**Figure 2:**
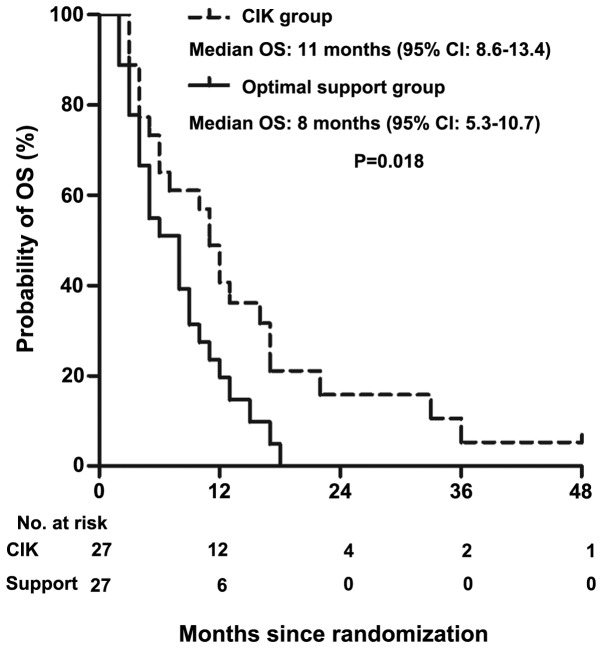
Kaplan-Meier curves for OS in advanced NSCLC patients following treatment with allogeneic CIKs and optimal support. The paired method was performed on patients with pathologically confirmed stage IIIb-IV NSCLC, and the 54 patients were divided into two groups; the allogeneic CIK (dashed line) and optimal support (solid line) groups. OS was defined as the survival time of patients from the date of enrollment to the date of mortality. OS, overall survival; NSCLC, non-small cell lung cancer; CI, confidence interval; CIKs, cytokine-induced killer cells.

Between January 2008 and December 2010, a total of 54 patients were recruited for the present study. In the allogeneic CIK and optimal support groups (n=27 per group), no marked differences were identified among age, gender, KPS score and pathological type or stage (P>0.05). In the allogeneic CIK and optimal support groups, the duration of follow-up was between four and 18 and six and 24 months, the median duration of follow-up was 13.0 and 14.0 months and the median survival was 11.0 (95% confidence interval (CI), 9.2–11.8 months) and 8.0 (95% CI, 9.2–11.8 months) months, respectively (χ^2^=5.618; P=0.018; Fig. 2).

## Discussion

The results of the present study demonstrated that treatment with autologous CIKs and haploidentical blood-induced CIKs exhibited few adverse events, which were characterized by insomnia and fever. No marked differences were identified in the occurrence of these side-effects between the two groups. The frequency of infusions, number of CIKs used for the infusion and repeated infusions over an extended period of time exerted no influence on the occurrence of adverse events. For stage I–IIIa lung cancer patients receiving an infusion of allogeneic CIKs, immune rejection was predicted to increase as the immune function in these patients was normal. However, compared with the advanced lung cancer patients and those treated with autologous CIKs, immune rejection in these patients with normal immune function did not increase. Of note, four patients treated with CIKs that were collected from the peripheral blood obtained from two offspring did not develop evident adverse events. These observations indicated that haploidentical blood-induced CIKs were safe, which provides support for the clinical application of these types of CIKs. This is particularly true for cancer patients who have exhausted their therapeutic options and whose autologous blood has failed to produce a sufficient number of CIKs for treatment, as well as for those who do not tolerate blood cell separators well. This approach is also highly convenient, as this method is easy to employ in combination with other strategies, which is almost impossible to achieve in treatment with autologous CIKs.

Although the offspring of patients are convenient blood donors for CIKs, viral infection or other reasons may render the collection of blood from these donors infeasible. In addition, *in vitro* culturing takes approximately three weeks to reach the number of CIKs and high proportion of CD3^+^CD56^+^ cells that is required. These considerations limit the use of this approach for the timely treatment of patients. Certain studies have attempted to obtain CIKs from umbilical cord blood (16,17). To the best of our knowledge, mononuclear cells that have been isolated from umbilical cord blood during a single collection do not provide a sufficient number of CIKs; in addition, the use of umbilical cord blood involves certain complex clinical issues. For patients with hematological tumors, allogeneic CIKs have been applied in the treatment of graft versus leukemia following stem cell translation (18–20). To extend this strategy across major HLA barriers in solid tumors, further studies on CIK alloreactivity are required. A previous study (21) involving CIKs indicated that CD3^+^CD56^+^ and CD3^+^CD8^+^ cells have distinct functions. CD3^+^CD56^+^ cells have almost no role in immune rejection, however, preserve the killing effect on autologous and allogeneic cancer cells, whereas, CD3^+^CD8^+^ cells are shown to be responsible for immune rejection. If methods are found that improve stimulation and induce the proliferation of CIKs to generate a sufficient number of cells for treatment, the separation of CD3^+^CD56^+^ cells may be beneficial for patients with diverse requirements. The results of the current study and those of previous studies (22–25) have demonstrated that dendritic cells increase the number of CIKs as well as the specific antitumor effect of CIKs following induction. In addition, the immortalization of CIKs following long-term *in vitro* stimulation is another issue. According to the correlation between telomerase and cancer cells, the environment of these cells was mimicked in the present study for CIK induction and infusion (26). The telomerase activity, proliferation, cell cycle progression and the antitumor effect of CIKs were subsequently detected dynamically. The results showed that telomerase activity reached a maximal level at 48 h following culture, and decreased and remained at a low level. However, at 45–60 days following culture, the majority of CIKs became necrotic or apoptotic.

Determining the efficacy of immune therapy remains a challenge. The new immune-related response criteria (27) for the determination of therapeutic efficacy provide some information, however, problems remain regarding the clinical application of these criteria. In addition, these criteria must be standardized, refined, validated and accepted. In numerous previous studies, the observations of immunological examination following CIK treatment were conflicting (3). Certain studies have shown that the percentages of CD3^+^, CD3^+^CD8^+^ and CD3^+^CD56^+^ cells were significantly increased following CIK treatment (28). In the present study, no changes in variables correlated with the cellular immunity, which may be attributed to the selection of the time points that were used for detection. The same time point that was used to determine the efficacy of chemotherapy was applied, which was four weeks following two cycles of treatment. In addition, whether the number of CIKs influenced the efficacy remains unclear. In the four cancer patients in the present study who received two continuous cycles of CIK treatment, only a transient increase in T lymphocytes and their subsets was observed. The IgG levels were found to continuously increase, thus, immune rejection or humoral immune enhancement requires further elucidation. In addition, the recent clinical detection methods used for cellular immunotherapy do not reflect change in the CD3^+^CD56^+^ subset, which may affect judgment of the immune function. Overall, the proportion of these immune cells in the peripheral blood and humoral immunity do not accurately reflect changes in immune function following CIK treatment (2,3).

Notably, similar to treatment with autologous CIKs (8), treatment with haploidentical CIKs also prolongs the survival of patients with advanced NSCLC. However, previous studies have failed to demonstrate an improved OS (1,9). Therefore, additional studies with larger sample sizes and proper design are required (8–11). Furthermore, the number and activity of CIKs and the proportion of CD3^+^CD56^+^ cells remains under investigation with regard to CIK treatment. In the present study, the number of CIKs used was comparable between the autologous and allogeneic groups, which may be attributed to the selection of patients. The autologous group included patients who had early-stage cancer, those who were in the rehabilitation stage following other treatments, or those that had refused other treatment options and received CIKs as their initial treatment.

In conclusion, the results of the current study indicated that haploidentical CIKs appear to be safe and prolong survival in the cancer patients described in the present study. However, appropriate donor selection and a non-intravenous route of infusion may be more effective, particularly for the regional therapy of patients with malignant pleural effusion, ascites or pericardial effusion, which requires further study.

## Figures and Tables

**Table I:** General information of the cancer patients receiving cytokine-induced killer cell therapy.

Characteristic	Autologous group	Allogeneic group	P-value
Patients, n	133	170	
Age, years			
Mean	62	64	0.082
Range	29–92	29–92	
Gender, n			
Male	71	92	0.293
Female	62	78	
Histology, n			
Lung cancer	40	54	0.665
Colorectal cancer	11	20	
Renal carcinoma	17	18	
Breast cancer	15	13	
Gastric cancer	12	11	
Other cancer	38	54	

**Table II:** Number of therapy cycles with autologous or allogeneic CIKs.

Cycles of CIK therapy	Patients, n	

	Autologous group	Allogeneic group
<4	114	151
4	7	10
5	2	4
6	4	1
7	0	1
8	3	2
>10	3	1
Total	133	170

CIKs, cytokine-induced killer cells.

**Table III:** Comparison of laboratory assessments of cancer patients prior to and following allogeneic CIK therapy (mean ± standard deviation; n=148).

	Allogeneic CIK therapy	
	
Variable	Prior to	Following
Routine blood test		
WBC, ×10^9^/l	6.60±2.14	6.78±1.44
RBC, ×10^12^/l	4.16±0.52	3.96±0.58
Hb, g/l	122.28±15.32	116.73±16.18
Platelet, ×10^9^/l	224.95±57.15	284.6±113.61
Liver function test, U/l		
AST	23.63±12.76	27.21±17.35
ALT	27.19±41.12	24.55±22.60
Kidney function test, μmol/l		
Nitrogen urea	8.4±15.7	4.84±1.37
Creatinine	72.45±21.69	74.78±14.71

P>0.05 following vs. prior to therapy. CIKs, cytokine-induced killer cells; WBC, white blood cells; RBC, red blood cells; Hb, hemoglobin; AST, aspartate aminotransferase; ALT, alanine aminotransferase.

**Table IV:** Comparison of immune function in cancer patients prior to and following allogeneic CIK therapy (mean ± standard deviation; n=132).

	Allogeneic CIK therapy	
	
Variable	Prior to	Following
Humoral immunity, g/l		
IgG	11.98±3.60	11.29±3.16
IgA	2.20±0.80	2.18±0.89
IgM	1.16±0.62	1.16±0.83
C3	1.23±0.30	1.23±0.24
C4	0.39±0.12	0.41±0.13
Lymphocyte subset, %		
CD3^+^	62.32±8.45	64.51±8.84
CD3^+^CD4^+^	34.76±7.87	32.74±8.67
CD3^+^CD8^+^	27.71±6.63	33.93±8.96
CD4^+^/CD8^+^	1.32±0.30	1.22±0.56
CD3^−^CD16^+^CD56^+^	25.20±5.57	19.78±4.34

P>0.05 following vs. prior to therapy. CIK, cytokine-induced killer cell.
